# “Cattle Welfare Is Basically Human Welfare”: Workers' Perceptions of ‘Animal Welfare' on Two Dairies in China

**DOI:** 10.3389/fvets.2021.808767

**Published:** 2022-02-08

**Authors:** Maria Chen, Daniel M. Weary

**Affiliations:** Animal Welfare Program, Faculty of Land and Food Systems, University of British Columbia, Vancouver, BC, Canada

**Keywords:** ethnography, animal welfare, qualitative methods, dairy farming, one welfare, livestock, employee, China

## Abstract

‘Animal welfare' (动物福利) is a foreign term in China, and stakeholder interpretations can affect receptiveness to the concept. Our aim was to explore workers' perceptions of animal welfare on two dairies in China. We used a mini-ethnographic case study design, with the first author (MC) living for 38 days on one farm and 23 days on a second farm. MC conducted semi-structured interviews (*n* = 13) and participant observations (*n* = 41) with farm management and staff. We used template analysis to generate key themes from the ethnographic data. Responses revealed a connection between human and animal welfare. Workers saw human welfare as a prerequisite to animal welfare, and cattle welfare as potentially mutually beneficial to humans. Some workers also saw an ethical obligation toward providing cattle with good welfare. Though some workers were unfamiliar with the term ‘animal welfare,' in daily practice caring for cattle led farm workers to ponder, prioritize, and make decisions relevant to welfare including lameness, morbidity, and nutrition. Workers in management positions appeared to embrace evidence-based animal care improvements, especially those which were perceived to also benefit people. Based on our findings, we suggest animal welfare initiatives should (1) consider worker welfare, (2) clearly communicate the concept of ‘animal welfare,' (3) identify mutual benefits, and (4) provide pragmatic, evidence-based strategies to improve welfare.

## Introduction

What does ‘animal welfare' mean to you? Your answer will likely vary depending on contextual factors including personal experience, culture, education and more. As Fraser (1) points out, animal welfare is a science informed by societal concerns and ethics. The term ‘animal welfare' was translated into Chinese in the 1990s, as ‘动物福利' [动物 meaning animal; 福利 meaning welfare; (2)], and it is not commonly used in China. You et al. (3) found that two thirds of Chinese public surveyed had never heard of this term. Indeed, the term may be problematic as ‘welfare' can sometimes be associated with receiving extra benefits beyond basic needs (4); when welfare is interpreted as additional benefits, some may deem a focus on animal welfare to be inappropriate in a context where human issues are still being addressed (5). That said, perceptions of animals and animal welfare are changing in Chinese society due to urbanization, pet ownership, increased media coverage and other factors (6, 7).

China is currently the world's largest livestock producer, home to a quarter of the world's terrestrial livestock (8). The welfare of these animals is impacted by increasing intensification of the livestock industry (9). To improve farm animal welfare in China, it is critical to engage with and understand the perspectives of stakeholders (e.g., farm managers, farm workers). Researchers have found that Chinese livestock stakeholders view animal welfare as important (10–12). While researchers have found that some Chinese livestock stakeholders report an intention to improve animal welfare (13), stakeholders also find it difficult to implement specific improvements (e.g., providing quality bedding, reducing painful procedures) (11). These earlier studies used pre-established definitions of animal welfare (12, 14), or researcher-generated categories (e.g., bedding, painful procedures) (11), which may fail to reflect the priorities of participants. Platto et al. (11) suggested that further qualitative research is needed to understand why some farmers perceive animal welfare as important but difficult to implement. Ethnographies (a set of methods used to understand social and cultural worlds), involve immersive fieldwork and participating in the daily lives of participants (participant observation) (15). As such, ethnographies are well-suited for gaining nuanced insight of insider perspectives in their local context.

Like many Chinese agricultural sectors, the dairy industry has experienced rapid restructuring, transitioning away from backyard farming to large, intensive farm systems (16), especially following the food safety scandals in mid-2000s which led government, industry, and consumers to shift focus from food security to food safety (17). Barkema et al. (18) noted that intensification in the dairy industry resulted in both welfare challenges and potential benefits in the terms of improved management and technical expertise. It is important to understand dairy farm workers' perceptions and interpretations of animal welfare, as this may influence the way they prioritize animal care practices (19).

We conducted an ethnographic case-study (for details see Section 2.2) of two Chinese dairy farms to understand farm workers' perceptions of animal welfare and animal care priorities.

## Materials and Methods

### Ethics

This study was approved by The University of British Columbia's Behavioral Research Ethics Board under #H18-03664. The first author MC obtained written consent from each farm's legal representative in order to access their farm. Participants within each farm were debriefed on the study, and verbal consent was obtained prior to participant observation and interviews (See Section 2.4 for details on data generation). For participants who agreed to participate but did not wish to participate in audio-recorded interviews, MC took notes during informal interviews. Farms and participants were anonymized to prevent identification. Farm are anonymized as Farm A and B, while participants are identified using a code (e.g., A1 is participant 1 from Farm A).

### Mini-Ethnographic Case Study

To understand workers' perceptions of animal welfare and their priorities in animal care, MC conducted a mini-ethnographic case study on two Chinese dairy farms (20). Ethnographic methods can improve credibility of findings, as researchers spend extended time participating in and observing people's daily activities, and data is triangulated from a variety of sources (observations, personal experiences, interviews with multiple individuals) (21). Findings from ethnographies are context specific. China is geographically diverse, and our findings are not intended to be generalizable, but some results may be relevant to similar contexts.

### Farm Context

We used purposive sampling to select two large-scale dairies in China (herd size >500 cattle) (16) since farm workers on these farms are best able to answer our research question, and farm access was feasible (22). We first provide some basic farm context important to understanding the results (information are accurate as of November 2019).

Farm A (herd size ~1500 Holstein cattle; ~50 workers) is in central China and owned by a private dairy processing company, while Farm B (herd size ~11,000 Holstein cattle; ~200 workers) is in east China and is owned by a state-owned dairy company. Cattle on both farms are housed in indoor stalls year-round, with several barns on each farm having access to fenced outdoor loafing areas. Lactating cows are milked twice daily on Farm A and three times a day on Farm B.

Farm A is older (built in the 2000s), and though infrastructure was considered “state-of-the-art” when built, it has not received significant new investments in recent years. Farm B is newer (built in the mid-2010s) and employed workers who were, on average, younger than those on Farm A (the average age of worker on Farm A was ~45 vs. ~30 on Farm B). Farm B's workers were also more educated on average (on Farm B, there was two Master's graduates, 25 trade school graduates, and more than 30 high school graduates; on Farm A there were 3 Trade school graduates and more than 10 high school graduates).

In terms of worker compensation, wages on Farm A were self-reported to be between 2,000 and 10,000 RMB/month (278–1389 EUR/month) (23). On Farm B, wages were displayed publicly and ranged between 2,900 and 11,000 RMB/month (403–1528 EUR/month) (23). For context, the minimum wage for the cities where Farm A and B were located was 1,600 and 1,550 RMB/month (222 and 215 EUR/month) (23), respectively, in 2019 (24). Employee benefits on both farms included worker dormitories and food provided in canteens. Farm B also included employee entertainment and gifts at the end of the year, while Farm A had a monthly celebratory meal. MC personally found the living conditions better on Farm B, including individual toilets, free worker uniforms and better food in the canteen.

### Data Generation

MC lived on Farm A from to September 27 to November 4, 2019, followed by Farm B from November 11 to December 3, 2019.

Each day, MC conducted participant observations, ethnographic interviews, and critical reflections to generate fieldnotes. Participant observation involved MC participating in the daily activities of her participants to understand common practices on these farms. Ethnographic interviews took place during participant observation and are informal but purposeful conversations focused on the research question and daily activities. In conversations, MC spoke in Mandarin while the participant used their preferred Chinese dialect. Participant observations and ethnographic interviews were useful for understanding daily animal care practice, and for generating discussion based on observed practices. For example, when MC was participating in herding, she asked “Why are we herding this way? Do you think the cattle like this?” to understand rationale behind practices and participant priorities.

MC conducted participant observation and ethnographic interviews with 20 workers on Farm A, including those in the farm management team (farm manager, deputy farm manager, 2 veterinarians, reproduction specialist, nutritionist), and members of the 5 farm teams (maternity, calf and heifer, milking parlor, reproduction, waste management). On Farm B, participants included 21 workers from 6 out of 8 departments (data collection, veterinary, maternity, calf and heifer, milking parlor, reproduction). The two remaining departments (nutrition, waste management) were excluded due to lack of time.

13 out of the 41 participants described above also agreed to participate in semi-structured, audio-recorded interviews (7 on Farm A; 6 on Farm B). Interviews focused on worker background, what cattle liked/disliked, what it meant for cattle to live a good life, and what they perceived of the term ‘animal welfare' (See Interview Guide in Appendix A).

### Data Analysis

Audio from the 13 interviews was transcribed verbatim by MC. Data from participant observations, ethnographic interviews, and critical reflections resulted in fieldnotes. All data (transcripts and fieldnotes) were organized in NVivo 12.6.0 (QSR International, Vancouver, Canada). MC used Template Analysis (a type of Thematic Analysis) (25) to analyze all data, with the goal of developing ‘themes' (overall patterns and conclusions) relevant to the research topics of how workers perceived ‘animal welfare,' and factors which were important to a cattle's good life.

MC completed the steps of Template Analysis (25): 1. Data familiarization through transcribing interviews and re-reading all transcripts and fieldnotes. 2. Initial ‘coding' of a subset of data (5 sections of fieldnotes and interviews rich in data relevant to the research topic). ‘Coding' involves labeling section of data with a ‘code' (e.g. ‘Improve milk quantity'). 3. Creating an initial ‘template,' a table which clusters similar codes together into categories; for example, ‘Improve milk quantity' and ‘Improve milk quality' fell under the ‘parent code' of ‘Farm benefits', under the larger ‘theme' of ‘Animal welfare benefits humans.' 4. Refining the template by coding the rest of the data using the initial template, while constantly adjusting the template to reflect MC's interpretation of the data. This resulted in the final template. MC also generated findings through a reflection document identifying data that appeared similar, different, contradictory, or surprising.

### Data Representation

Through data analysis, MC generated 4 themes relevant to how workers perceived animal welfare (see Appendix B for Final Template), and 3 themes relevant to what workers thought was important to a good life for cattle (see Table 1 for simplified template).

**Table 1:** A compilation of workers' responses to what is important to cattle, and what cattle like.

**Animal factors**	
Health	Adequate nutrition (e.g., quality and quantity of feed, milk, colostrum, water)
	Less disease and injury (e.g., lameness, mastitis, metritis)
Emotions	More positive emotions (e.g., being happy, music, massage)
	Less stress (e.g., humane handling; less human disturbance)
	Less pain (e.g., euthanasia, pain relief)
Natural living	Freedom (e.g., ability to walk around, access outdoors, enjoy sunshine)
	Relationships (e.g., keep calves together, mother and calf together)
**Environment factors**
Tangible infrastructure	Stall comfort (e.g., bedding, stall dimensions, number of stalls)
	Flooring (e.g., milking parlor, walkways, free-stalls, and outdoors)
	Equipment (e.g., cattle chute, milking equipment, headlock)
Intangible environment	Weather (e.g., temperature, humidity)
	Cleanliness (e.g., biosecurity measures, hygiene)
**Human factors**
Employee management	Organizational culture (e.g., establish good norms and values)
	Improve worker competence (e.g., education, training, attract and retain high-performing workers)
	Implement incentive systems (e.g., merit-based income, praise and recognition)
	Human welfare (e.g., accommodation, wage, work conditions)
Stockmanship	Humane handling (e.g., no hitting or prodding; gentle interactions)
	Love (e.g., be empathetic; put cattle first)

Themes are illustrated using relevant fieldnote or transcript excerpts. Since the research question focuses on worker perceptions of animal welfare, most findings are based on quotations from semi-structured interviews and unstructured ethnographic interviews during participant observation. Comments on worker behaviors and human-cattle interactions are included in certain instances to provide context. Participant observations were essential to understanding worker priorities and behaviors.

## Results

We begin by providing some further context for the two farms, and then describe the workers' conceptions of animal welfare using 4 themes, focusing specifically on how animal welfare was connected to worker welfare. Lastly, we present a descriptive summary of aspects of animal care that workers considered important, and how workers prioritized aspects of animal care.

### Context

The intensification of the dairy industry was experienced by workers on both farms. Many workers on both farms used to work for smaller farms, either as owners or as veterinarians or reproductive specialists servicing a region.

Workers identified how this transition affected their motivation for raising cattle, and the quality of animal care. Farm A's reproductive specialist (A8; who had previously worked for small farms) shared the following:

“Large farms focus on economic profit, previously people raised cattle because they liked cattle! But for cattle health, large farms have the advantage. Nutrition, veterinary care, reproductive care, small farms cannot achieve this. You can't afford the equipment. For cattle, there is more personalized care on small farms, the families love their cattle deeply (心疼牛). But in terms of productivity, management, of course they are inferior to the large farms.”

This quote reflects the perception that owners of small farms may raise cattle with more affection, but improved management and expertise on large farms can result in better cattle health and productivity outcomes.

Changing farm size also affects farm ownership and worker demographics. Unlike the owners of small dairy farms, the farm workers on these large farms did not have complete decision-making authority over animal care. The large farms have organizational structures like other businesses: managers and technical workers on both farmers were paid more and had more decision-making power (e.g., department leaders on Farm B and the farm management team on Farm A). Managers and technical workers sometimes worked their way up from more entry-level positions. A member of the farm management on Farm A (A2) explained, “farm workers [not in management positions] normally have no education. Like milkers, they are usually local, earning around 2,500–3,000 [RMB/month]. [In contrast], the management and technical workers are hired from all over the country.” Conversations with multiple workers confirm that most of the lower paid workers were local, while higher paid workers were more likely to be hired from other provinces.

There were more worker complaints on Farm A, where wages were perceived and reported to be low. One worker on Farm A (A7) shared “People are only allowed to keep working, like machines are only allowed to keep running.” The local, lower paid workers (such as milkers, barn staff, animal caretakers) on Farm A referred to themselves as “受苦的” (those who suffer/endure hardships). A lower paid milker on Farm A (A4) commented in frustration: “I have elders and young ones (上有老下有小) to take care of… I have no option but to do this work!” On both farms, the most common reason for local workers to do such work was because it is “close to home,” though on Farm B a veterinarian (B19) shared it was because of “high employee welfare.”

### Perceptions of ‘Animal Welfare'

Some workers on both farms felt that “animal welfare” was an unfamiliar term, though those in management and technical positions were more likely to have heard it. Themes emerging from discussion of the workers' perception of animal welfare included: 1. Human welfare as prerequisite for animal welfare, 2. Animal welfare as mutually beneficial to humans, 3. Animal welfare means additional benefits, and 4. Humans have ethical obligations to cattle.

#### Human Welfare as a Prerequisite for Animal Welfare

From MC's observations it appeared that both worker welfare and quality of animal care were higher on Farm B. According to one worker (B2) on Farm B, “they recently improved our meal plan, everyone is more motivated to work! Cattle welfare is basically human welfare, only when human lives are improved then they can take good care of the cattle.” As a participant observer, MC can also attest that her motivation to work improved significantly on the second farm where her diet and living conditions were better.

MC observed that several lower-paid workers on Farm A treated the cattle roughly, but members of the farm management team consistently treated the cattle gently. One member of the management team (A8) explained: “servicing the people, servicing the cattle, that's all non-sense, you know, our subsistence comes first… If you paid me only 3,000 [RMB/month], I'd mistreat the cattle too.” This quote suggests that one reason the management team was more patient was that they perceived their subsistence needs to be met.

One member of the barn staff (A17), one of the lower paid, local workers on Farm A, jokingly expressed discontent when comparing his welfare to that of the cattle, as reflected in MC's fieldnotes:

It is 5 AM, I walk with worker A17 through the foggy morning air as the cattle quietly feed on their total mixed rations in the barn.

A17: “What do you study again?”

MC: “I study animal welfare, dairy cattle welfare. Have you heard of it?”

A17: “Dairy cattle welfare, it's living well, being happy! What do you think?”

MC: “Mhmm.”

A17 laughs: “Here the cattle get treatment when they are ill, drink whenever they want, eat whenever they want… Cattle welfare, there isn't even human welfare! I don't even live like cattle!”

MC: “Do you think the cattle live well-here?”

He yells excitedly: “Their life is top quality! The boss here has hundreds of thousands of RMB… These cattle live like children in the cities! Their life is like the emperors.”

This worker defined ‘animal welfare' as “living well, being happy,” focusing on the cattle's subjective experience. Despite problems on Farm A, this worker perceived cattle welfare to be “top quality.” This perception was likely based on comparison with his own welfare: the cattle's treatment included medical and nutritional care, aspects he perceived to be lacking in his own life.

A member of Farm A's management team (A2) suggested how this way of thinking may be prevalent and might even be associated with resistance to change:

“Many [animal care] issues are caused by the local “backwards thinking” (思想落后). For example, with animal welfare, people say “humans don't even have welfare why give animals welfare?” they don't understand [the rationale for improving animal welfare].”

#### Cattle Welfare as Mutually Beneficial to Humans

While some workers saw human welfare as influential to shaping animal welfare, some also saw how improving cattle welfare benefits humans. These benefits focused on health, productivity, and profit, but also included safer interactions, and improved worker wellbeing.

Several workers from both farms equated animal welfare as the provision of basic necessities. In Chinese, ‘yi shi zhu xing' (衣食住行) means ‘basic necessities' (the phrase literally translates to food, clothes, shelter, transport). This phrase was used by a few participants on both farms to describe both human and cattle welfare. Departmental leader B17 shared:

“[Animal welfare] is the same as for humans. “Yi shi zhu xing.” Yi [clothes] refers to temperature. Whether I am cold in the winter or hot in the summer. Zhu [shelter] refers to the stalls. Are my living quarters clean […] are my bedding comfortable and dry? If I am not comfortable then I will not lie, if I stand for a long time my milk yield is bound to decrease. And then food, is it enough for me to produce milk […] Then xing [transport] is walking, is the ground flat, is it slippery. Will I fall? Will I injure my hooves?”

In this example, the participant identified a variety of environmental aspects related to animal care that shapes the experience of cattle. He used his conception of basic needs of humans (food, clothes, shelter, transport) as a reference point to articulate the needs of the cattle. He also identified how the cattle's experience, such as comfort, is tied to important economic considerations such as milk yield.

A member of Farm A's farm management team (A12) also conceptualized cattle welfare as basic necessities that are also beneficial to humans. On Farm A, cattle care was relatively poor prior to the improvements brought on by the arrival of the new management team in March, 2019 (6 months before MC's arrival). In a talk to the farm workers in a monthly farm meeting A12 stated:

“Chen is studying animal welfare. Human welfare is “food, shelter, transport.” And entertainment. Cattle [welfare] is “food, shelter, transport, milking” (吃住行挤) […] All our work needs to revolve around the cattle. Cattle welfare, don't see them as livestock (畜生), see cattle as your partner in earning, when they are comfortable, they will produce more, and we will benefit. […] our first step was to fix the farm, next we are going to set a base for achieving the cattle's basic needs (food, shelter, transport, milking). We will standardize our procedures. Humans must correct our mistakes to the cattle. We need to be responsible, not sloppy, and fix issues starting from our ideology.”

In this definition, the participant recognized the similarities between human and cattle welfare, while suggesting that entertainment is important to humans but not cattle. He viewed cattle welfare as in alignment with productivity goals and further viewed humans to have an ethical responsibility to provide good animal care.

A member of Farm B's reproductive team (B20) also shared this sense of moral responsibility toward cattle: “Do you believe in karma (因果报应)? […] When you act with bad intentions, there will be bad consequences. It is the same with hitting and scolding cattle. She will be nervous, anxious, and her reproductive rate will decrease, and she could kick you.” This worker provides an ethical framework for linking intentions and actions with their consequences, highlighting how poor treatment of cattle leads to detrimental outcomes for both cows and workers.

Apart from farm profitability benefits, those who were attached to their cattle also benefited emotionally from the wellbeing of their animals. A veterinarian (A5) from Farm A was consistently caring and gentle toward his calves. When MC asked what he felt when he was with the calves, he said: “When I see them in a good mood, I am happy.”

Overall, aspects of animal welfare, such as the provision of basic necessities was perceived to be in alignment with management and farm profit goals, and some workers saw it as their moral obligation to provide good animal care.

#### Additional Benefits for Cattle

The term ‘welfare' used in a human context (e.g., 员工福利, employee welfare) can mean additional benefits provided on top of basic needs. Further conversation with the barn staff on Farm A (A17), suggested that he interpreted ‘welfare' this way:

MC: “What do you mean by “human welfare” (人的福利)?”

A17: “Human welfare is giving you a worker uniform, or at the end of the year giving you some oil.” (Employee welfare in Chinese companies is used to attract and maintain workers, and these can include providing employee meals, worker uniforms, insurance, or gifts such as cooking oil)

MC: “I think cattle welfare is letting cattle live a good life (好的生活), what do you think is important to cattle?”

A17: “Cleanliness, listening to music, showering. Being clean.”

This quote suggests that human welfare was perceived to mean something given to workers in addition to their basic needs such as wage. This may explain why he also included “listening to music” as something important to cattle welfare. Interestingly, departmental leader B15 also mentioned giving cattle music as a way to improve animal welfare. He referred to rumors about Japanese Wagyu beef cattle whose superior meat quality was thought to result from “music, beer, and massages” (a misconception sometimes appearing in both Chinese and non-Chinese media) (26, 27). If ‘animal welfare' was understood to mean luxuries such as “music, beer, and massages,” it is understandable that some workers viewed welfare improvements as unnecessary or impractical for their farms, especially when they perceived human welfare needs as unmet.

#### Humans Have a Moral Responsibility

Due to their relationship to cattle, some workers appeared to view welfare as a moral responsibility owed to the animals. Farm B's reproductive team was the highest performing department within all the farms in Company B (measured using indicators such as high reproduction rate, low calf mortality). MC noticed the members appeared to be respectful toward cattle, partly due to the attitude of the departmental leader (B7). In a daily meeting, he introduced MC to his team:

“This is Chen Rong Shan. She studies animal welfare. She studies the humanities. This is also a natural science, searching for the laws of nature (自然规律). She is here to study about macroscopic issues related to a cattle's thoughts and experiences. This includes anything related to cattle. Currently humans are the strong, the rulers. Because of this, we need to find the way of nature, respect nature. It is a crime to be disrespectful. Only when you give a man power, you can see their true nature. Give a man a prod, a stick. You can immediately see what kind of person they are. We need to consider humans, but also cattle. Don't harm her because you are angry. Work with her using your brain, not brute force. In the beginning, cattle didn't like to be disturbed by humans. We first violated (侵犯) her freedom and captured her. Now we need to take responsibility. Cattle have souls. Just because we cannot see it does not mean it doesn't exist. Consciousness is in alignment with Darwin's theory of evolution. We cannot anger her soul.”

In his conception of welfare, animal welfare is the understanding of cattle experience using social and natural sciences. He appeared to value cattle intrinsically, suggesting that humans have the responsibility to provide cattle good care, in part because he considered cattle as beings with souls who are able to communicate with and understand humans emotionally (有灵性; a view shared with workers on both farms including A5, A6, A7, A9, A20, and B20). MC did not inquire about the religions of the participants, but she did not observe any religious practices. Departmental leader B7 conceived humans as dominant over domesticated animals, explaining that this results in an ethical obligation to provide humane treatment and good care.

### Providing a Good Life: Practicalities and Priorities

Though not all workers were familiar with the term ‘animal welfare,' when asked to comment on what they thought was important to cattle, all were able to articulate this based on their daily work. Workers brought up a variety of factors, which we categorized in Table 1 into three overlapping areas: animal-based factors, environment-based factors, and human-based factors.

Worker responses generated a diverse list of factors important to cattle, but in practice most found it was unreasonable or impractical to address all these needs. For animal-based factors, workers generally prioritized health and biological functioning since this was perceived to directly align with productivity goals, for example through addressing nutrition (e.g., colostrum management), disease (e.g., mastitis) and injuries (e.g., lameness). Workers identified mental wellbeing and natural living as important to cattle, but focused on affective states (e.g., reducing stressful handling) and natural living (e.g., providing outdoor access to calves on Farm A) only when this was also perceived to improve health and biological functioning. Pain relief was not prioritized as it was not viewed to improve productivity (e.g., a veterinarian on Farm A was unreceptive toward using pain medication as it was perceived to increase costs without productivity benefits). Additionally, farm profit was prioritized when cattle were culled to generate some income from their sale, rather than euthanizing them on farm. Access to the outdoors was seen as important, but farm management on Farm A felt that well-managed indoor systems provided better comfort and health benefits to cows.

Environmental factors were identified as important but, depending on an individual's decision-making power, difficult to change. For example, on Farm A infrastructure improvements required approval and investment from the company. As such, even though farm management and workers identified the need to improve equipment (e.g., hoof trimming chute and headlock) and infrastructure (e.g., barn and stall design), these changes were not approved. There were also logistical challenges. For example, workers on both farms identified sand as the ideal bedding but recognized it was hard to access sand in their region. Additionally, environmental legislation that required farms to install manure recycling infrastructure meant that Farm B switched to using recycled manure as for bedding.

Human factors, especially employee management, were identified on both Farms A and B as among the most important factors shaping animal care [see companion paper Chen et al. (22)]. Though aspects like organizational culture (especially company culture) were found to be difficult to change, farm management attempted to motivate workers to improve animal care using merit-based income and by attempts to improve conditions for farm workers. While humane handling was recognized as important in reducing stress and increasing milk yield, some workers on Farm A were observed to treat cattle roughly. Hoof trimming staff on both farms reported that they wanted to treat cattle gently but felt that gentle handling would not enable them to get their work done as there were too many cattle to treat and cattle were moving too slowly.

## Discussion

### Perceptions of ‘Animal Welfare'

Although the science of animal welfare is now well-established (28, 29), prominent scholars argue that providing a single definition of the term ‘animal welfare' is difficult [e.g., Duncan (30)]. Some individuals in China view ‘animal welfare' as a Western concept (31), potentially out of step for a region where human welfare needs are not fully addressed (32). For example, in a context where food safety issues are still a concern, it can appear tone-deaf to talk about ‘animal welfare,' especially if a connotation of this term is additional provisions on top of basic needs (e.g., giving cattle music); such perceptions can lead to resistance and even ridicule (5). However, as Cao (5) argues, the concept of animal welfare is compatible with traditional Chinese values of Confucianism, Taoism, and Buddhism, all of which promote compassion toward animals. Thus, clearer communication with stakeholders about the underlying concept of animal welfare may facilitate engagement.

Promisingly, several workers in this study showed positive attitudes toward animal welfare; they saw animal welfare as beneficial to the animal's health and productivity, as well as to the worker's job satisfaction. Identification of such mutual benefits may be key to engaging Chinese livestock stakeholders in animal welfare improvements (33).

Consistent with our findings, Sinclair et al. (34) found that profit was a key motivator for stakeholders to improve animal welfare. However, the relationship between animal welfare and profit is complex. In certain situations, where animal welfare is so poor that productivity is compromised, improving welfare will likely lead to better health, productivity, and profit. Research has indicated that reducing lameness (35) and improving humane handling (36) can improve productivity. However, high milk production can also be associated with health disorders in dairy cattle (37). Efforts to improve animal welfare should identify evidence-based strategies that improve animal welfare and are economically sustainable for farms (for example by finding markets for high welfare products) (38).

Farm worker reported worker welfare as key to ensuring animal welfare; interviews with dairy farmers in Finland (39) and Denmark (40) also showed this association. This finding is in alignment with the concept of “One Welfare,” where animal welfare and human welfare are recognized as intimately connected (41, 42). One Welfare is already promoted within the Chinese farm animal welfare field, for example at the 2nd World Conference on Farm Animal Welfare held in China (43).

Worker attitudes and values can be influenced by factors such as farm size, species, and production systems (44). Yang (45) found that senior producers in the Chinese egg sector were familiar with the term ‘animal welfare.' However, their definition of this term often focused on biological functioning at the cost of mental wellbeing and natural living, resulting in negative views of cage-free systems.

Yang et al. (46) found that Chinese fish stakeholders were also familiar with the term ‘animal welfare,' although some felt that this concept referred more to terrestrial animals, and dissimilarities between fish and humans made fish welfare less of a concern [see also Miralles et al. (47)]. In contrast, workers in this study described a variety of factors important to cattle, including physical and mental health, natural living, environmental conditions, and the caregivers involved. These extra dimensions may have emerged because cattle are perceived as more similar to humans, but likely also because of the time MC spent with the workers, proving opportunities to understand what they thought were important to cattle. Workers in this study likely had more contact with the cattle and more opportunity to know individual animals compared to those working with poultry and fish.

### Providing a Good Life

During discussions with participants, using the term “animal welfare” seemed to result in a focus on more conceptual issues, whereas talking about what cattle liked and disliked, what was important to cattle, and what gave cattle a good life, resulted in more focus on the daily care of cattle and more practical issues. This difference may explain why Sinclair et al. (13) found that Chinese industry stakeholders reported high intention and even confidence in improving ‘animal welfare,' while Platto et al. (11) found that Chinese farmers perceived it difficult to address specific management challenges such as “keeping the animals and barns clean.”

The areas of concern identified in the current study were consistent with those identified in the English animal welfare literature, including the three-sphere concept of health, mental wellbeing, and the ability to live a natural life (48), and concerns about humane handling (49). In practice, workers seemed to prioritize concerns related to biological functioning, likely because farm profitability was seen as non-negotiable [see Anneberg and Sandøe (40)].

The current study illustrates the benefits on on-farm ethnography in describing farm worker perspectives. Specifically, the immersive fieldwork and extended time spent with the participants allowed a more in-depth exploration of what workers thought of cattle, cattle care, and ‘cattle welfare.' By focusing on the participants own words and actions, we attempted to understand their conception of ‘animal welfare,' what it means to provide cattle a good life, and how animal care is prioritized in practice.

Current animal welfare research is often mono-disciplinary, focusing on either the animal's perspective or the people's perspectives (50). Taylor and Hamilton (51) suggest that future studies should combine approaches to better understand both the experience of animals and the perspectives of human stakeholder responsible for animal care. Such studies may be able to further our understanding of the complex interplay between human and animal welfare on farms.

### Implications

Based on our findings, we make the following suggestions to improve welfare on farms with similar contexts. Firstly, our results illustrate the importance of ensuring worker welfare in efforts to promote the welfare of animals under their care. Secondly, our results suggest that the meaning of ‘animal welfare' can be misunderstood, creating a barrier to change. Adopting more commonly understood language such as “what is important to the animal” or “what the animal prefers” may result in better understanding by all stakeholders. Thirdly, approaches that benefit both animals and farm workers should be identified and addressed (e.g., adopting high welfare practices that also improve profit or worker safety). Lastly, pragmatic, evidence-based strategies to improve welfare should be provided to both decision makers (e.g., managers), and frontline workers (e.g., animal care staff).

## Conclusions

Workers saw human welfare as a prerequisite to animal welfare, and cattle welfare as potentially beneficial to humans. The relationship between cattle and humans also meant some workers viewed improving cattle welfare as their ethical obligation. Though some were unfamiliar with term ‘animal welfare,' caring for cattle led farm workers to ponder, prioritize, and make decisions relevant to specific aspects of ‘animal welfare' such as lameness, morbidity, reproduction, nutrition. Participants appeared pragmatic in their approach, and willing to embrace evidence-based animal care improvements that benefited both themselves and the cattle.

## Data Availability Statement

The data presented in this study are available upon reasonable request from the corresponding author. The data are not publicly available to protect the confidentiality of the participants, and due to the personal and subjective nature of the data.

## Ethics Statement

This study was approved by the University of British Columbia's Behavioral Research Ethics Board under #H18-03664. The Ethics Committee waived the requirement of written informed consent for participation.

## Author Contributions

MC and DW: conceptualization. MC: methodology, software, investigation, data curation, writing—original draft preparation, and funding acquisition. DW: writing—review and editing and supervision. All authors contributed to the article and approved the submitted version.

## Funding

This research was funded by Open Philanthropy and Good Ventures.

## Conflict of Interest

The authors declare that the research was conducted in the absence of any commercial or financial relationships that could be construed as a potential conflict of interest.

## Publisher's Note

All claims expressed in this article are solely those of the authors and do not necessarily represent those of their affiliated organizations, or those of the publisher, the editors and the reviewers. Any product that may be evaluated in this article, or claim that may be made by its manufacturer, is not guaranteed or endorsed by the publisher.
