# Supplementary material for: “Cattle Welfare Is Basically Human Welfare”: Workers' Perceptions of ‘Animal Welfare' on Two Dairies in China
Source: Front Vet Sci. 2022 Feb 8;8:808767. doi: 10.3389/fvets.2021.808767 (PMC8861200; doi:10.3389/fvets.2021.808767)
Supplement: Supplementary file 1 [file Table_1.DOCX]

Supplementary Material

## Appendix A

**Appendix A.** Interview Guide

| **Topic** | **Question** | **Translation** |
| --- | --- | --- |
| Demographics | 你的老家是哪里的？ | Where is your hometown? |
|  | 你来场上干了多久？ | How long have you been working here? |
|  | 你为什么来这里/这个岗位工作？ | Why are you working here/in this position? |
| Cattle well-being | 对牛来说，什么是好的生活? | For cattle, what is a good life? |
|  | 什么对牛很重要? | What is important to cattle? |
|  | 牛喜欢/不喜欢什么? | What do cattle like/dislike |
|  | 你听过“动物福利”吗? | Have you heard of “animal welfare”? |

## Appendix B

**Appendix B.** Final template for workers’ perceptions of animal welfare

| **Theme** | **Parent code** | **Child code** |
| --- | --- | --- |
| Worker welfare needed 保障员工福利 (WW) | WW-Worker welfare  员工福利 (WK) |  |
|  |  | WW-WK-Wage 工资 |
|  |  | WW-WK-Holiday 假期 |
|  |  | WW-WK-Gifts 礼物 |
|  |  | WW-WK-Bonus 绩效工资 |
|  |  | WW-WK-Entertainment 娱乐 |
|  |  | WW-WK- Meals 伙食 |
|  |  | WW-WK-Living conditions 住宿 |
|  |  | WW-WK-Worker uniform 工衣 |
|  | WW-Higher worker welfare  提高员工福利 (HI) |  |
|  |  | WW-HI-Worker welfare important 重要性 |
|  |  | WW-HI-Attract workers 吸引员工 |
|  |  | WW-HI-Retain workers 保留员工 |
|  |  | WW-HI-More work motivation 有动力 |
|  |  | WW-HI-Gentle treatment of cattle  温和对待牛 |
| Animal welfare benefits humans  动物福利的益处 (AW) | AW-Basic needs of cattle  牛的基本需求 (BN) |  |
|  |  | AW-BN-Basic needs 衣食住行 |
|  |  | AW-BN-Cattle comfort 奶牛舒适度 |
|  | AW-Farm benefits  农场的益处 (FB) |  |
|  |  | AW-FB-Cattle as earning partner  挣钱的伙伴 |
|  |  | AW-FB-Improve profit 利润 |
|  |  | AW-FB-Reduce cost 成本 |
|  |  | AW-FB-Improve cattle health 奶牛健康 |
|  |  | AW-FB-Improve reproduction 繁育 |
|  |  | AW-FB-Improve milk quality 奶的质量 |
|  |  | AW-FB-Improve milk quantity 产奶量 |
|  | AW-Workers benefits  员工的益处 (WB) |  |
|  |  | AW-WB-Improve worker safety 员工安全 |
|  |  | AW-WB-Merit-based income 绩效工资 |
|  |  | AW-WB-Human cattle relationships  人牛关系 |
|  |  | AW-WB-Worker wellbeing 员工感受 |
| Welfare as additional benefits  福利是额外的添加 (AB) | AB-Human welfare as extra benefits 人的福利 |  |
|  | AB-Cattle welfare as extra benefits 牛的福利 (CW) |  |
|  |  | AB-CW-Shower 洗澡 |
|  |  | AB-CW-Music 音乐 |
|  | AB-Resistance to 'animal welfare' 抵触‘动物福利’ (RE) |  |
|  |  | AB-RE-Humans don't live like cattle  人不如牛 |
|  |  | AB-RE-'Backwards thinking 落后思想 |
| Ethical obligation to cattle  对牛的道德责任 (EO) | EO-Cattle are valuable  牛本身很重要 (VA) |  |
|  |  | EO-VA-Prioritize cattle over self  优先照顾牛 |
|  |  | EO-VA-Everything for cattle 一切为了牛 |
|  |  | EO-VA-Like/love cattle 喜欢奶牛 |
|  |  | EO-VA-Have feelings for cattle  对牛有感情 |
|  |  | EO-VA-Consider cattle feelings  考虑牛的感受 |
|  |  | EO-VA-Poor cow 可怜的牛 |
|  |  | EO-VA-Cattle emotionally connects to humans 通人性 |
|  |  | EO-VA-Cattle have soul 灵性/灵魂 |
|  | EO-Humans responsible for good cattle care  人有责任照顾好牛 (HR) |  |
|  |  | EO-HR-Feel responsible to cattle 有责任 |
|  |  | EO-HR-Karma 因果报应 |
|  |  | EO-HR-Humans violated cattle's freedom  侵犯奶牛 |
|  |  | EO-HR-Humans need to correct mistakes  为牛改错 |
|  |  | EO-HR-Humans domesticated cattle  驯化奶牛 |
